# Factors influencing UK medical students’ choice of general practice: a systematic review

**DOI:** 10.3399/BJGP.2025.0226

**Published:** 2026-06-01

**Authors:** Catharina Savelkoul, Lydia Prieto i Sepúlveda, Sophie Park, Nia W Roberts, Stavros Petrou, Joan E Madia, Simon de Lusignan, Catia Nicodemo

**Affiliations:** 1 Nuffield Department of Primary Care Health Sciences, University of Oxford, Oxford, UK; 2 Erasmus School of Health Policy & Management, Erasmus University Rotterdam, Rotterdam, the Netherlands; 3 Bodleian Health Care Libraries, University of Oxford, Oxford, UK; 4 Royal College of General Practitioners, London, UK; 5 Brunel University of London, Business School, London, UK

**Keywords:** career choice, general practice, medical education, primary health care

## Abstract

**Background:**

There are currently concerns about recruitment to UK general practice. There have been various efforts and approaches to increase recruitment to general practice, and there is a lack of contemporary insights and knowledge about the factors that shape medical students’ career intentions.

**Aim:**

To identify and analyse the key factors influencing UK medical students' choice of general practice as a career pathway.

**Design and setting:**

This was a systematic review of empirical literature about factors influencing career choice in UK undergraduate medical education synthesising both quantitative and qualitative evidence across UK medical education contexts.

**Method:**

A systematic review was conducted following the PRISMA guidelines. Systematic searches of seven electronic databases (Medline, Embase, PsycINFO, ERIC, Web of Science, British Education Index, and EconLit) were conducted to identify primary research published from 1990 to 2024. The Bland–Meurer theoretical framework structured the analysis.

**Results:**

The systematic review identified 29 studies. Three critical dominant factors emerged: an educational disconnect between GP recruitment needs and medical curricula; the persistent negative hidden curriculum experienced by students in various settings; and the important role of authentic clinical placements and positive role models in challenging negative stereotypes.

**Conclusion:**

The findings from this review suggest that medical education structures and institutional cultures influence medical students’ decisions about general practice careers. Medical schools and policymakers can improve recruitment by addressing the educational factors that shape career choice. Increasing high-quality general practice exposure in the curriculum, actively countering negative perceptions of general practice, and promoting positive GP role models are all crucial.

## How this fits in

The UK faces a projected shortage of approximately 15 000 GPs by 2036/2037, with a declining proportion of UK medical graduates pursuing general practice. Previous research has identified various contributing factors but lacked a contemporary synthesis within a coherent theoretical framework. This systematic review examines factors influencing UK medical students’ career decisions, finding three dominant influences: curricula that inadequately represent general practice, a persistent negative hidden curriculum, and the impact of clinical placement quality. The study’s revised Bland–Meurer model incorporates these findings, providing a comprehensive framework to improve GP recruitment.

## Introduction

The UK faces a projected shortage of approximately 15 000 GPs by 2036/2037.^1^ The near future looks potentially more concerning: a 2024 survey by the Royal College of General Practitioners indicated that over 40% of GPs are likely to leave the profession within 5 years.^2^ This exists against a backdrop of increasing healthcare demands in primary care, driven by an ageing population with complex multimorbidity and a growing emphasis on community-based care.^3–5^

In response, Health Education England has mandated that 50% of all new medical graduates enter general practice.^6^ However, the number of UK medical graduates pursuing general practice has declined in recent years; annual intakes are becoming increasingly dependent on international medical graduates.^7^ The percentage of international medical graduates in GP training rose from 34% in 2019 to 52% in 2023.^7^

The proportion of UK foundation year 2 doctors appointed to GP training programmes decreased from 36.1% in 2012 to 31.6% in 2019.^8^ Subsequent data indicate a further decline.^9^ With current recruitment levels well below the target of 50%, understanding what drives these career choices becomes crucial.^6^ As the Wass report notes, *‘students do not choose general practice by chance’*.^10^

This raises the questions: what factors influence UK medical students’ decisions about pursuing careers in general practice? And what theoretical frameworks best explain the complex interplay between individual, institutional, and systemic factors in medical career decision making? To answer these questions, a systematic literature review was conducted to synthesise the evidence on which factors influence UK medical students’ decisions about pursuing careers in general practice.

## Method

### Search strategy

Working in close consultation with a specialist medical librarian, the authors of the current study developed and iteratively refined a search strategy across seven electronic databases: Medline via OvidSP, Embase, PsycINFO, ERIC, Web of Science Core Collection, British Education Index, and EconLit. The search strategy incorporated both controlled vocabulary (MeSH terms and Embase subject headings) and free-text keywords, structured around four conceptual domains: medical education, general practice, career choice, and the British healthcare context (Supplementary Table S1). The systematic review protocol was prospectively registered with Open Science Framework (DOI: 10.17605/OSF.IO/KZC5A) before data extraction.^11^

### Selection criteria

Studies were included based on prespecified criteria developed through consensus. Eligible studies examined career decision-making processes among UK medical students, with particular emphasis on general practice as a specialty choice. Primary empirical research published from 1990 to October 2024 was included, corresponding with the implementation of the NHS and Community Care Act 1990. Included study designs encompassed quantitative methods, qualitative methods, and mixed methods.

Studies were excluded if they:

- focused exclusively on postgraduate trainees or fully qualified doctors;
- examined exclusively non-UK contexts; and
- lacked empirical data; or
- investigated specialty choice without consideration of general practice.

Although the primary focus was on factors influencing career choice, studies were also included that evaluated teaching interventions if they reported outcomes related to general practice career intentions.

### Data extraction and quality assessment

Two reviewers independently extracted the data. Recognising the limitations of the PICO (population, intervention, comparison, and outcome) framework for qualitative research synthesis, the SPIDER framework (setting, population, intervention, design, evaluation, research type) was used to inform data extraction tables and categories through a customised Excel extraction form.^12–14^ This approach better accommodated the heterogeneous nature of the evidence base, particularly for capturing qualitative findings about students’ career decision-making processes.

Quality assessment employed validated tools appropriate to study design: the JBI Critical Appraisal Checklist for Analytical Cross-Sectional Studies for quantitative studies, and the CASP checklist for qualitative research (see Supplementary Information S1).^15,16^

### Synthesis framework

The Bland–Meurer model of determinants of primary care specialty choice (1995) provided the theoretical framework for systematically categorising the findings. The model identifies three principal domains:

- student characteristics (personality traits, socioeconomic background, and personal values);
- specialty characteristics (perceived prestige, work–life balance, and professional opportunities); and
- medical school influences (curriculum design, clinical exposure, and institutional culture).^17^

## Results

**Figure 1.**
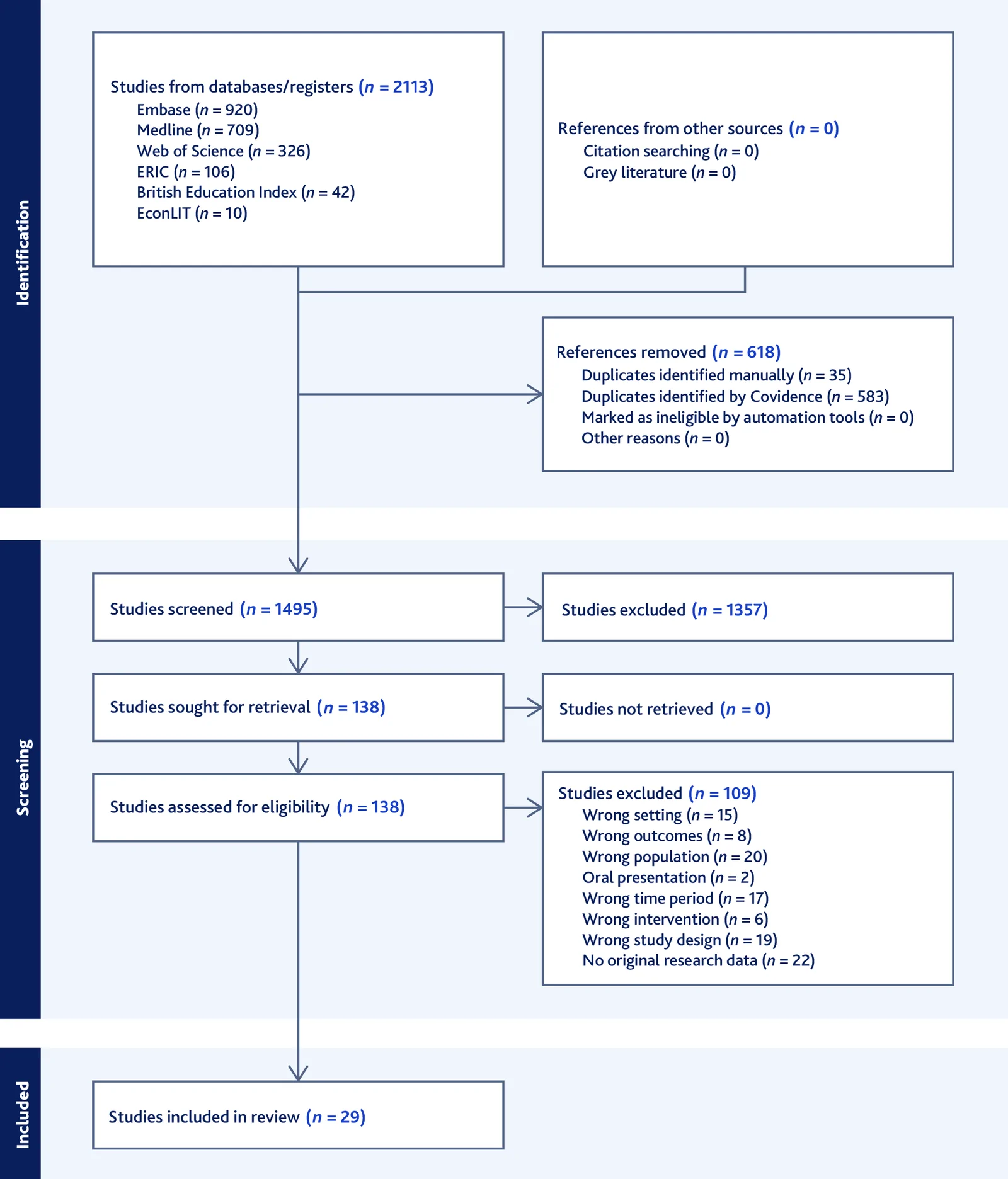
PRISMA flow diagram.

The initial electronic database searches yielded 2113 citations. After removing 618 duplicates, 1495 unique papers were screened. Title and abstract screening excluded 1357 citations that failed to meet the inclusion criteria. The remaining 138 papers underwent full-text review, resulting in 29 studies that met all eligibility criteria (Figure 1).^18–46^

Quality assessment indicated most studies had a low risk of bias and appeared in peer-reviewed journals. Quantitative studies generally demonstrated strong sampling representativeness, particularly in large-scale surveys and longitudinal cohorts. Qualitative studies scored well on the CASP checklist. A full summary of quality appraisal results is presented in Supplementary Tables S2–4.

Table 1 summarises the factors the review identified as influencing medical students’ decisions towards GP careers. These are categorised by:

- student characteristics (dominant domain);
- specialty characteristics (dominant domain);
- medical school influences (dominant domain); and
- external influences (minor domain).

Detailed information on the methodology and key findings for each of the 29 included studies is provided in Supplementary Table S5.

**Table 1.** Factors influencing medical students’ decisions towards general practice careers: evidence synthesis through the Bland–Meurer theoretical framework

Domain and factor	Evidence from systematic review
**Student characteristics**	
Personal values and priorities	
Preferences for part-time options	Sinclair *et al* (2006);^20^ Lambert *et al* (2012);^18^ Gami and Howe (2020);^19^
Preference for long-term patient relationships	Hogg *et al* (2008);^22^ Cleland *et al* (2012);^21^
Preference for holistic care	Hogg *et al* (2008);^22^ Gami and Howe 2020^19^
Demographic factors	
Gender (female students more likely to choose general practice)	Henderson *et al* (2002);^23^ Goldacre *et al* (2007);^24^ Drinkwater *et al* (2008);^46^ Cleland *et al* (2012);^21^ Gami and Howe (2020)^19^
Graduate-entry status	Goldacre *et al* (2007)^24^
Age and maturity	Carlin *et al* (2021)^25^
Personality traits	
Preference for variety versus specialisation	Edgcumbe *et al* (2008);^27^ Turner *et al* (2021)^28^
**Specialty characteristics**	
Professional attributes	
Misconceptions about prestige	Mattsson *et al* (1991);^31^ Barber *et al* (2018);^29^ Reid and Alberti (2018)^30^
Misconceptions about intellectual challenge	Hogg *et al* (2008);^22^ Chellappah and Garnham (2014);^32^
Variety of work	Edgcumbe *et al* (2008);^27^ Gami and Howe (2020)^19^
Career structure	
Training pathway (shorter, more structured)	Edgcumbe *et al* (2008)^27^
Flexibility and part-time options	Hogg *et al* (2008);^22^ Lambert *et al* (2012)^18^
Career progression opportunities	Nicholson *et al* (2016)^33^
Research opportunities	Darnton *et al* (2021);^34^ Misky *et al* (2022)^35^
Work conditions	
Perceived work–life balance	Hogg *et al* (2008);^22^ Cleland *et al* (2012);^21^ Lambert *et al* (2012)^18^
Workload and administrative burden	Parekh *et al* (2021);^36^ Hook *et al* (2024)^37^
Professional isolation	Edgcumbe *et al* (2008);^27^ Parekh *et al* (2021)^36^
Remuneration	Edgcumbe *et al* (2008)^27^
**Medical school influences**	
Curriculum factors	
Exposure to GP placements	Alberti *et al* (2017);^38^ Amin *et al* (2018)^39^
Placement quality	Allsopp and Taggar (2018);^40^ Nicholson *et al* (2016)^33^
Placement timing (early and longitudinal better)	Howe and Ives (2001);^41^ Amin *et al* (2018)^39^
Curricular representation (underrepresented)	Vaidya *et al* (2019)^42^
Role models and mentorship	
GP tutors	Mattsson *et al* (1991);^31^ Nicholson *et al* (2016);^33^ Parekh *et al* (2021)^36^
Role model impact (positive and negative)	Henderson *et al* (2002);^23^ Edgcumbe *et al* (2008)^27^
Institutional culture and hidden curriculum	
‘GP bashing‘/institutional attitudes towards GPs/the ‘They say’ phenomenon	Firth and Wass (2007);^43^ Rodriguez *et al* (2015);^45^ Nicholson *et al* (2016);^33^ Reid and Alberti (2018);^30^ Carlin *et al* (2021);^25^ Darnton *et al* (2021);^34^ Banner *et al* (2023)^44^
Teaching Interventions	
Structured career tutorial	Allsopp and Taggar (2018)^40^
Specialty-specific approaches	Allsopp and Taggar (2018);^40^ Gami and Howe (2020)^19^
Clinical independence during placements	Nicholson *et al* (2016)^33^
**External factors**	
Changing perceptions over time	Morrison and Murray (1996);^26^ Sinclair *et al* (2006);^20^ Gami and Howe (2020)^19^
External context (for example, COVID-19 impact)	Hook *et al* (2024)^37^

### Student characteristics

#### Personal values and priorities

Students who value continuity of care were twice as likely to prefer general practice.^21^ Two studies found that many students were attracted to the holistic nature of GP consultations.^19,22^ Across various studies, students who valued a good work–life balance and part-time opportunities had a preference for general practice.^18–20^

#### Demographic factors

Gender emerged as a significant predictor of interest in general practice careers. Multiple studies found that female students were significantly more likely to express a preference for GP careers.^19,21,23,24^ ^46^ Age (graduate-entry status) also influenced career preference because general practice was chosen by more students from the graduate-entry route than from the non-graduate entry route.^24^ Another study suggested that maturity and prior life experiences shaped students’ career interests. Participants noted that students who entered medical schools at a younger age and those in the early years of medical school were ‘quite impressionable’ and therefore more susceptible to the impact of negative comments, whereas graduate-entry route students and those in later years were more confident in their career aspirations and less influenced by such views.^25^ In contrast, one study found that students with previous degrees or intercalated degrees were less likely to choose general practice.^26^

#### Personality traits

Students’ orientation towards clinical variety or specialisation appeared closely aligned with underlying personality dispositions.^27^ Those who enjoy breadth and unpredictability were more inclined towards careers in general practice.^27,28^

### Specialty characteristics

#### Professional attributes

The variety of work in general practice and the continuity of care were identified as a positive influence on GP career intention.^19,27^ However, perceptions and attitudes misrepresenting the professional attributes (intellectual challenge and societal importance) of general practice remain a negative influence on GP career intention.^10,30–32^

#### Career structure

The findings from the included studies suggest that students were attracted to the shorter, more structured training pathway into general practice.^27^ Flexibility and part-time options were often noted here, particularly for those considering family responsibilities.^16,20^ However, three studies found that the misconception that there are fewer research opportunities in general practice negatively influences GP career intention.^33–35^

#### Work conditions

Work conditions in general practice have changed substantially.^10^ Older studies included in this review identified work–life balance as a major factor attracting medical students to general practice.^18,21,22^ More recent studies identified that concerns about workload and administrative burden have a negative influence on GP intention.^36,37^ Professional isolation was another concern, and two studies reported that some students feared being professionally isolated in general practice settings compared with hospital teams.^27,36^

### Medical school influences

#### Curriculum factors

Exposure to GP placements significantly influenced career choices. One study identified a significant correlation between the amount of exposure to general practice and GP career intention.^38^ Another found that longitudinal (extended, recurring over the academic year) GP placements were more effective than traditional block placements in increasing GP career intention.^39^ High-quality placements, characterised by authentic clinical exposure, meaningful responsibility, and positive role modelling, increased interest in general practice.^33,40,42^

The timing of placements matters; two studies included found that early and longitudinal exposure to primary care had a greater impact on career intentions than late, brief placements.^39,41^ One study demonstrated the systemic underrepresentation of general practice in many medical curricula; with students spending a median of just 8 weeks (9%) on GP placements, despite over 40% of Core Training Year 1 (CT1) and Specialty Training Year 1 (ST1) training posts being in general practice.^42^

#### Role models and mentorship

The experience with GP tutors was an important influence on career decisions in the included studies. Studies reported that positive placements with engaging GP tutors significantly increased interest in primary care.^27,33,36^ Across these studies, positive or high-quality GP placements were characterised by good supervision from GP tutors who were approachable and enthusiastic role models. The importance of such conditions is highlighted by one study that identified personal experience with GP tutors as the strongest influence on attitudes toward primary care.^23^

#### Institutional culture and hidden curriculum

The prevalence of a hidden curriculum in medical schools can be a barrier to choosing general practice as a career. Multiple studies have documented that students frequently heard negative comments about general practice from clinical teachers.^26,30,33,43,44^ Such denigration may take the form of overtly derogatory comments^25^ or through the absence of primary care perspectives in teaching^43^ and the implicit positioning of hospital specialties as more prestigious^30^ within the medical school. One study identified this as the ‘They say’ phenomenon defined as *‘a passive and pervasive perception, without a known source, whereby usually negative perceptions circulate around the undergraduate community’.* ^44^

#### Teaching interventions

Although a systematic assessment of all teaching interventions was beyond the scope of this review, the evidence identified suggests that structured educational interventions increase interest in general practice. For instance, paired careers tutorials, in which students explored general practice alongside another specialty in a structured, comparative format, increased the likelihood of choosing general practice.^40^

### Temporal and external influences

Several studies identified changing perceptions over time.^19,20^ Crises have been shown to shape GP career intentions; one study found that the COVID-19 pandemic fundamentally altered students’ views of general practice by associating it with increased remote consulting, reduced patient contact, greater professional isolation, and heightened workload pressures, compounded by negative media portrayals, which together reduced its appeal as a career choice.^37^

## Discussion

### Summary

**Figure 2.**
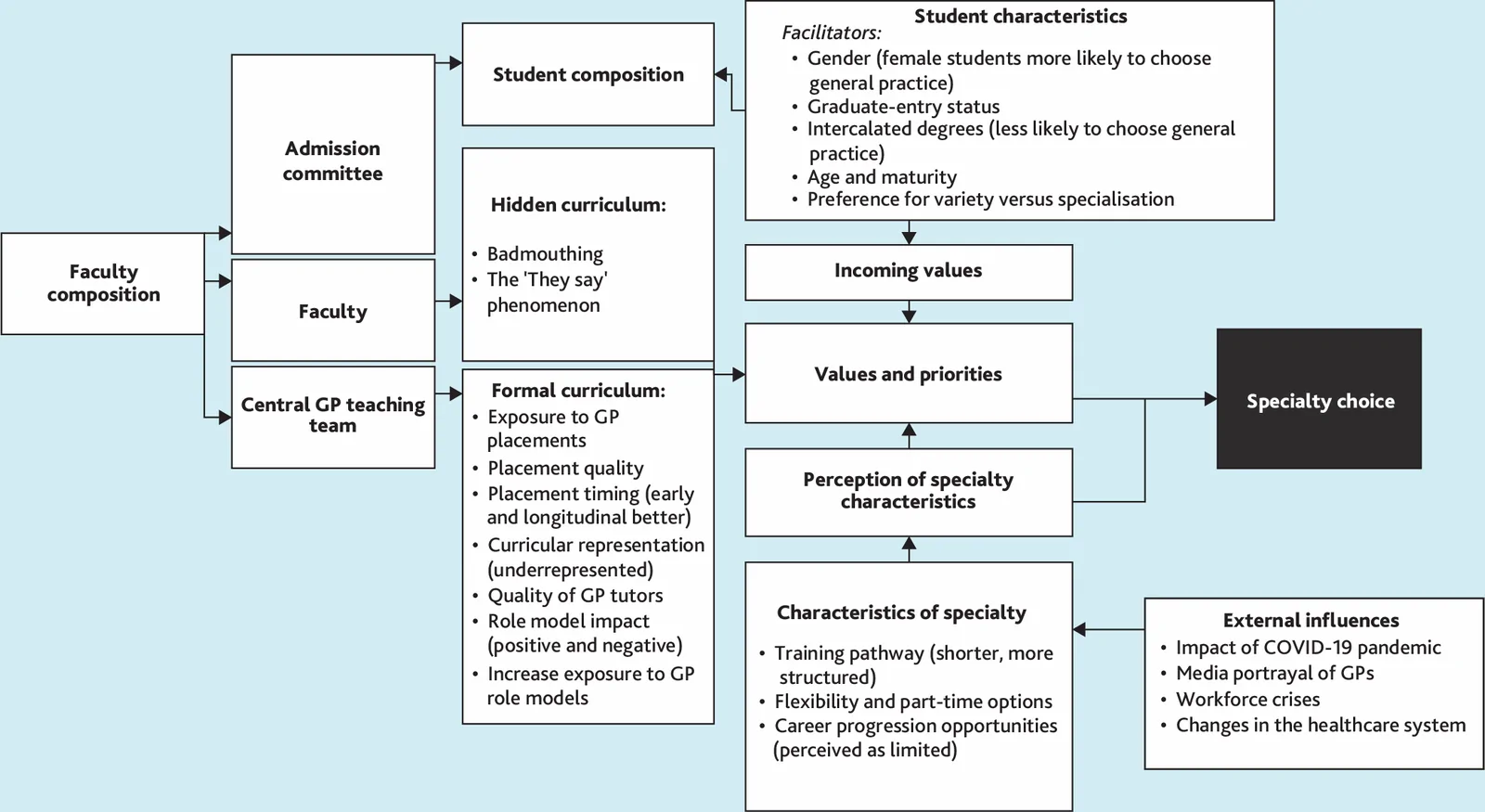
A revised model of factors influencing general practice career choice.

This systematic analysis, which applied the Bland–Meurer theoretical framework, found evidence for many factors influencing UK medical students’ choice of general practice as a career. Three dominant themes were constructed from the evidence synthesis (Figure 2).

First, student characteristics play a key role in predisposing individuals towards or away from general practice. Gender remains a significant predictor with female students showing stronger preference for careers in general practice.^21,23,25^ Individual factors interact with personality traits, with students who prefer variety and person-centred care more likely to consider general practice.^27,28^

Second, specialty characteristics significantly have an impact on medical students’ career decisions, with professional attributes and career structure influencing perceptions of general practice. Misconceptions around the relative prestige between general practice and hospital specialties have been cited as a factor negatively influencing career choice.^29–31^ Many students value the variety and intellectual challenge of the general practice.^19,27^ Although earlier studies identified perceived flexibility and work–life balance as incentives for choosing general practice,^18,22^ concerns about workload and administrative burden have been reported as negative influences.^27,36,37^

Third, medical schools have an important and varying effect on career decisions, with the (hidden) curriculum and role models shaping students’ perceptions of general practice. The quantity and quality of GP placements directly correlate with career interest^38–40^ and positive role models can significantly increase attraction to primary care.^23,27,33^ However, the persistent denigration of general practice within medical education, the ‘They say’ phenomenon, continues to undermine recruitment efforts.^26,30,33,43,44^

### Strengths and limitations

The primary strength of this review is the use of systematic methods to identify and synthesise evidence from diverse study designs to capture the multifaceted nature of career decision making.

A limitation of this review was the broad temporal scope of the included studies. Although the 1990 cut-off date corresponds with the implementation of the NHS and Community Care Act, the 35-year timespan limited the contemporary relevance of some findings. For instance, some of the included studies emphasised work–life balance as a major attraction of general practice.^18–20^ However, these findings were based on older evidence, and their contemporary relevance is limited given the substantial rise in workload reported in recent years.^1^ Another older study reported that students with previous or intercalated degrees were less likely to choose general practice.^26^ However, as that study was conducted nearly three decades ago, its findings may have limited relevance to the current context of medical education.

The authors were also limited in the causal inferences that can be drawn from these data. The cross-sectional designs and self-reported intentions inherent within many of the included studies introduce temporal ambiguity and reporting biases. Few studies employed multivariate analyses to control for confounding variables. Those that did often showed attenuated effect sizes after adjustment.^21,29^ Correlational statistics,^38^ low response rates,^19^ and institutional selection effects further limit causal interpretations. The absence of quasi-experimental designs or propensity score matching represents a notable methodological gap. This necessitates cautious interpretation.

### Comparison with existing literature

This study, to the authors’ knowledge, is the first UK-focused systematic review that aggregates evidence on how student, specialty, medical school, and other characteristics shape medical students’ intention to pursue a career in general practice. One scoping review synthesised international and Irish literature, and identified curriculum exposure, positive clinical rotations, role models, personal attributes, and community influences as key factors influencing GP career intention.^47^ Its scope did not fully reflect the specific factors influencing GP career intention within the context of UK medical education (such as differences between medical schools) and NHS context (such as increased GP workload and the projected shortages). By focusing exclusively on UK evidence, the findings are directly relevant and applicable to informing workforce policy, recruitment strategies, and medical education.

Furthermore, policy-focused work, most prominently the Medical Schools Council report *By Choice — not by Chance* and subsequent commentaries, has long called for: substantial expansion of GP teaching, longitudinal placements, and active antidenigration policies.^10^ The current findings substantiate each recommendation and add precision by:

- aligning curriculum time with workforce needs and ensuring placements are authentic (continuity, responsibility, supervision);
- monitoring and addressing the hidden curriculum; and
- supporting GP role models to counter misconceptions about intellectual challenge and research careers in general practice.

Figure 2 provides a revised theoretical model.

### Implications for research and practice

This evidence synthesis has implications for changes in medical education. First, medical schools should increase the proportion of curriculum time devoted to general practice, aiming to align this more closely with hospital specialties and, where possible, use longitudinal placements.^10,39,42^ The introduction of the harmonised undergraduate medical education and training tariff in 2022, which now provides equitable funding across all clinical settings, including general practice, may make expanding GP placements more feasible.^48^ This new funding arrangement may also facilitate improvements in quality such as better role modelling by GP tutors through improved remuneration.^33,49^

The evidence that medical students’ career intentions are shaped by the attitudes of those who teach and mentor them, and that denigration of general practice can discourage students from pursuing the specialty, demonstrates the importance of implementing the antidenigration policies recommended in the Wass report.^10^ In parallel, widening participation initiatives, by attracting students more likely to work in underserved areas, might offer an additional strategy to increase recruitment into general practice.^10,50^

Finally, to address the limitations in causal inference arising from reliance on cross-sectional and self-reported designs, and the limited use of controls for confounders in the quantitative studies, future quantitative research should consider longitudinal, quasi-experimental methodologies. The UK Medical Education Database presents an opportunity for this.^51^

## Data

All data supporting the findings of this review are available in the published studies cited in the reference list. The full data extraction summary is provided in Supplementary Table S5.
